# Paclitaxel mitigates structural alterations and cardiac conduction system defects in a mouse model of Hutchinson–Gilford progeria syndrome

**DOI:** 10.1093/cvr/cvab055

**Published:** 2021-02-24

**Authors:** Álvaro Macías, J Jaime Díaz-Larrosa, Yaazan Blanco, Víctor Fanjul, Cristina González-Gómez, Pilar Gonzalo, María Jesús Andrés-Manzano, Andre Monteiro da Rocha, Daniela Ponce-Balbuena, Andrew Allan, David Filgueiras-Rama, José Jalife, Vicente Andrés

**Affiliations:** 1 Vascular Pathophysiology Area, Centro Nacional de Investigaciones Cardiovasculares Carlos III (CNIC), 28029 Madrid, Spain; 2 CIBER en Enfermedades Cardiovasculares (CIBER-CV), Madrid, Spain; 3 Department of Internal Medicine, University of Michigan, Ann Arbor, MI 48109, USA; 4 Center for Arrhythmia Research, University of Michigan, Ann Arbor, MI 48109-2800, USA; 5 Department of Cardiology, Cardiac Electrophysiology Unit, Hospital Clínico San Carlos, 28040 Madrid, Spain; 6 Myocardial, Pathophysiology Area, Centro Nacional de Investigaciones Cardiovasculares Carlos III (CNIC), 28029 Madrid, Spain

**Keywords:** Hutchinson–Gilford progeria syndrome, Animal model of cardiovascular disease, Electrophysiology, Lamin A/C, Progerin, Cardiomyocytes

## Abstract

**Aims:**

Hutchinson–Gilford progeria syndrome (HGPS) is an ultrarare laminopathy caused by expression of progerin, a lamin A variant, also present at low levels in non-HGPS individuals. HGPS patients age and die prematurely, predominantly from cardiovascular complications. Progerin-induced cardiac repolarization defects have been described previously, although the underlying mechanisms are unknown.

**Methods and results:**

We conducted studies in heart tissue from progerin-expressing *Lmna^G609G/G609G^* (G609G) mice, including microscopy, intracellular calcium dynamics, patch-clamping, *in vivo* magnetic resonance imaging, and electrocardiography. G609G mouse cardiomyocytes showed tubulin-cytoskeleton disorganization, t-tubular system disruption, sarcomere shortening, altered excitation–contraction coupling, and reductions in ventricular thickening and cardiac index. G609G mice exhibited severe bradycardia, and significant alterations of atrio-ventricular conduction and repolarization. Most importantly, 50% of G609G mice had altered heart rate variability, and sinoatrial block, both significant signs of premature cardiac aging. G609G cardiomyocytes had electrophysiological alterations, which resulted in an elevated action potential plateau and early afterdepolarization bursting, reflecting slower sodium current inactivation and long Ca^+2^ transient duration, which may also help explain the mild QT prolongation in some HGPS patients. Chronic treatment with low-dose paclitaxel ameliorated structural and functional alterations in G609G hearts.

**Conclusions:**

Our results demonstrate that tubulin-cytoskeleton disorganization in progerin-expressing cardiomyocytes causes structural, cardiac conduction, and excitation–contraction coupling defects, all of which can be partially corrected by chronic treatment with low dose paclitaxel.

## Graphical Abstract

**Figure:**
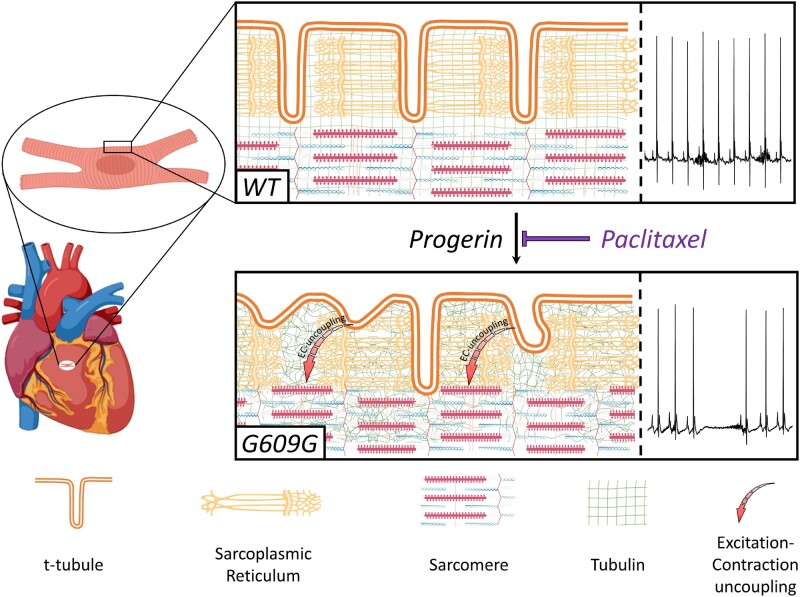

**Time for primary review: 43 days**

## 1. Introduction

The *LMNA* gene encodes A-type lamins (lamin A and lamin C), essential members of the nuclear envelope in mammals that play key structural and modulatory functions in several cellular processes, such as chromatin organization, signal transduction, and gene transcription, among others.^1^ Mutations in the human *LMNA* gene cause a group of diseases called laminopathies, including Hutchinson–Gilford progeria syndrome (HGPS), an extremely rare genetic disorder with an estimated prevalence of 1 in 18 million people (www.progeriaresearch.org) that is characterized by premature aging.^2^^,^^3^ HGPS patients typically appear normal at birth and do not exhibit symptoms until around 1–2 years of age. Then, they begin to exhibit failure to thrive and develop signs redolent of physiological aging, including atherosclerosis and arterial stiffness, leading to premature death at an average age of 14.6 years, mainly due to heart disease or stroke.^4^^,^^5^

Most HGPS patients carry a *de novo* heterozygous c.1824C>T (pG608G) point mutation in the *LMNA* gene.^6^^,^^7^ This mutation triggers usage of an unconventional 5’ splice donor site in exon 11 that results in removal of 150 nucleotides in *LMNA* mRNA, which causes the synthesis of a mutant protein called progerin. This truncated form of lamin A remains permanently farnesylated and accumulates in the nuclear envelope, causing severe alterations in nuclear structure and multiple cell functions.^2^^,^^3^ Several studies have also reported low levels of progerin expression in cells and tissues during normal aging (reviewed in References^3^,^8^). Remarkably, most hallmarks of normal aging also occur in progeria, suggesting a role of this mutant protein on some pathophysiological features commonly observed in the elderly population (e.g. cardiovascular alterations).^2^^,^^3^

Among cardiovascular alterations, our studies in HGPS patients^9^ and progeroid *Zmpste24^−/−^* mice,^9^*Lmna^G609G/G609G^* (G609G)^10^ mice and *LMNA* 1824 C > T minipigs^11^ revealed defective cardiac repolarization as a common feature of progeria. Specifically, HGPS patients showed progressive overt ST segment alterations as they age, which were also present in progeroid *Zmpste24^−/−^* mice^9^ and G609G mice.^10^ More careful sequential follow-up also showed that conduction abnormalities and bradycardia are present at late stages of the disease in both mice and pig models of progeria.^9–11^ However, the mechanisms by which progerin expression causes these and other cardiac alterations remain largely unknown. Recent data indicate that the cytoskeleton is connected to the nucleus through nesprin and SUN proteins, elements of the linker of nucleoskeleton and cytoskeleton (LINC) complex.^12^ The nucleoplasmic region of human SUN domain proteins binds to A-type lamins,^13^^,^^14^ and nesprins bind to the tubulin-cytoskeleton.^15^^,^^16^ Integrity of the LINC complex is critical for the maintenance of nuclear morphology^17^ and force transmission between the nucleus and cytoskeleton,^18^^,^^19^ which in turn is crucial for proper functionality of cardiomyocytes.^20^^,^^21^

In this study, we tested the hypothesis that progerin expression causes cardiac defects at least in part through alterations in the cardiomyocyte cytoskeleton. We used the progerin‐expressing G609G mouse model, which develops most features of progeria, including failure to thrive, lipodystrophy, bone abnormalities, and cardiovascular alterations.^22–24^ Our results identify tubulin-cytoskeleton disorganization in the progeroid heart as a mechanism contributing to defective electrical activity, including sinoatrial block and reduced intraventricular excitability. We also demonstrate that paclitaxel ameliorates structural and functional alterations in the G609G mouse heart, thus identifying the tubulin-cytoskeleton as a possible therapeutic target in HGPS.

## 2. Methods

Detailed material and methods are provided in the Supplementary material online.

### 2.1 Study approval

All animal experiments conducted in this study conformed to EU Directive 2010/63EU and Recommendation 2007/526/EC, enforced in Spanish law under *Real Decreto 53/2013*. The local ethics committees and the Animal Protection Area of the Comunidad Autónoma de Madrid (PROEX 050/18) approved all animal protocols.

### 2.2 Mice

Mouse experiments were carried out in 17–19 week-old male and female *G609G* mice^22^ (an age close to their maximum survival, see Supplementary *Figure S1*), and in age-matched wild-type (WT) littermates (all C57BL/6). Animals were reared and housed in accordance with institutional guidelines and regulations. G609G mice were housed with WT littermates to maintain the best conditions of housing. For *in vivo* experiments, animals were anaesthetized with 0.5–2% isoflurane in an inhalation chamber. Animals were euthanized in a CO_2_ chamber.

### 2.3 Pigs

Heart cross-sections were obtained from 4.3-month-old to 5.4-month-old progeroid heterozygous *LMNA* c.1824C > T Yucatan minipigs and age-matched WT controls were sourced from a previous study.^11^

### 2.4 Cardiomyocyte isolation

The procedure for cardiomyocyte isolation was adapted from Garcia-Prieto et al.^25^ Briefly, after euthanasia the mouse heart was cannulated through the ascending aorta, retrogradely perfused and enzymatically digested.

### 2.5 Immunofluorescence and 3D-reconstruction of cardiomyocytes

Isolated mouse cardiomyocytes were fixed and processed for confocal microscopy and image quantification.

### 2.6 Intracellular calcium transient measurements

Field stimulation was conducted at varying frequencies for 10 s separated by 15–20 s intervals to measure cytosolic Ca^2+^ dynamics.

### 2.7 Transmission electron microscopy

Small pieces (around 2 mm^2^) of left ventricle (LV) wall were fixed with 4% paraformaldehyde and post-fixed with 1% osmium tetroxide in phosphate-buffered saline for 1 h. Tissue blocks were then contrasted in 0.5% aqueous uranyl acetate. Ultra-thin (60 nm) sections were cut and contrasted with 2% aqueous uranyl acetate and Reynolds lead before image acquisition.

### 2.8 Cardiac function assessment by magnetic resonance imaging (MRI)

Images were obtained and analysed using a modified method previously described.^26^

### 2.9 Cardiac echocardiography

Transthoracic echocardiography was performed blind by an expert operator from CNIC Advanced Imaging Unit using a high-frequency ultrasound system (Vevo 2100, Visualsonics Inc., Canada) with a 40 MHz linear probe.

### 2.10 Histology and immunofluorescence studies

Mouse hearts were dehydrated, and embedded in paraffin for histological and immunofluorescence studies.

### 2.11 Western blot

Hearts were homogenized, lysed and protein extracts were stored at −80°C until use. For western blot, 30 µg of protein were loaded into SDP-PAGE gels. Protein abundance was normalized to the intensity of actinin used as loading control.

### 2.12 Electrocardiogram (ECG)

ECG recordings were acquired at 2 kHz using a MP36R data acquisition workstation (Biopac Systems) and exported with AcqKnowledge software (Biopac Systems) for automatic analysis with custom R and Matlab scripts.

### 2.13 Patch-clamping in isolated cardiomyocytes

We used standard patch-clamp recording techniques to measure electrophysiological parameters in isolated mouse cardiomyocytes.

### 2.14 Paclitaxel treatment

The 10-week-old G609G mice received for 9 weeks 3 i.p. injections per week of paclitaxel as previously described.^27^^,^^28^ For control, both WT and G609G mice received vehicle.

### 2.15 Statistical analysis

Statistical analyses were conducted in Prism-5. Between-genotypes comparisons were generally made by unpaired two-tail Student’s *t*-test. Unless otherwise stated, we used one- or two-way ANOVA with Tukey’s correction for comparison between more than two groups. Unless otherwise stated, *N*-number represents the amount of animals, and *n*-number the amount of samples used in each experimental set. Data are expressed as mean±SEM, and differences were considered significant at *P*<0.05.

## 3. Results

### 3.1 G609G mice exhibit cardiac functional and structural alterations

**Figure 1:**
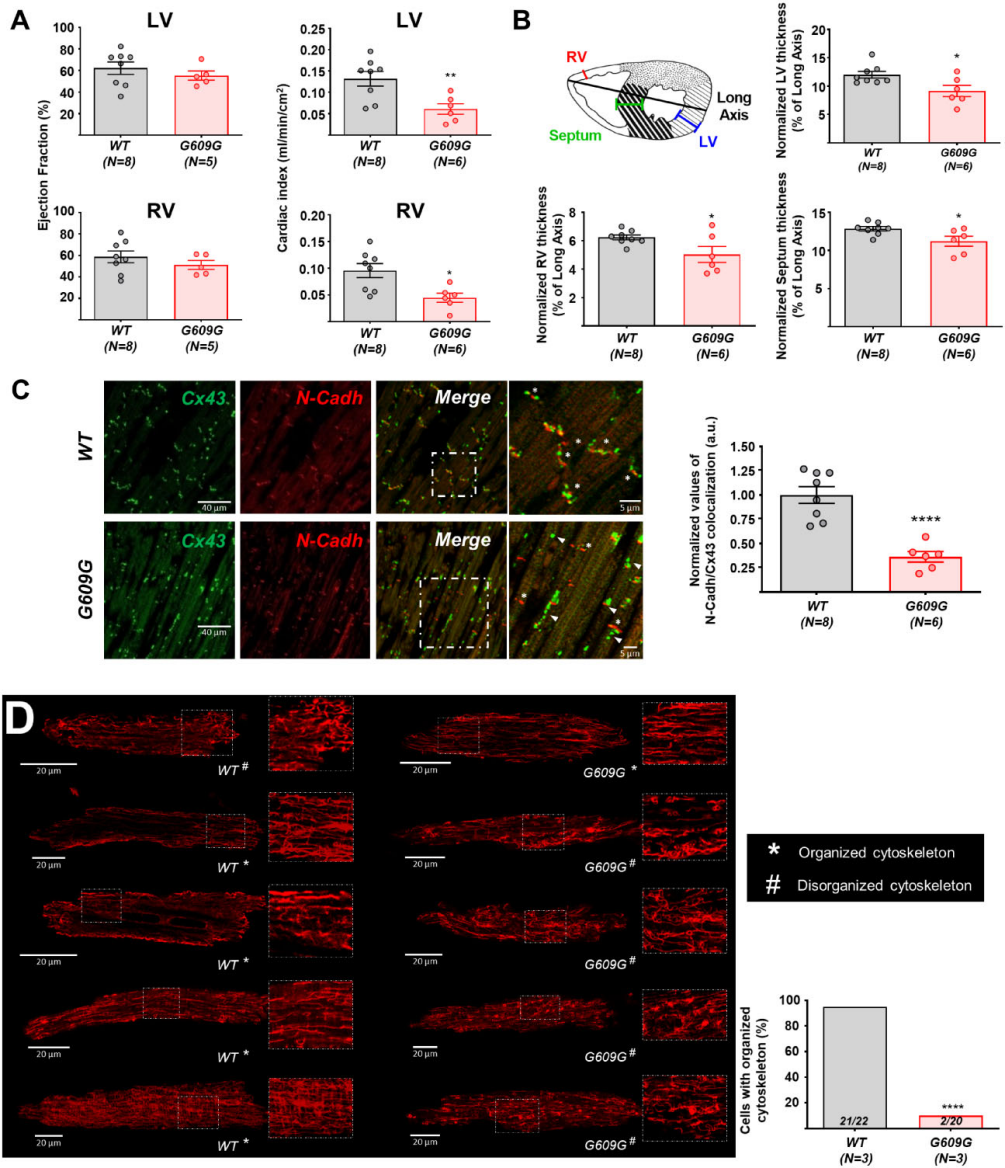
G609G mice show functional and structural cardiac alterations with disruption of the tubulin-cytoskeleton organization. (*A*) Ejection fraction and cardiac index determined by MRI in anaesthetized WT and G609G mice. (*B*) Cardiac thickening in the RV, LV, and septum of G609G mice, all normalized to the long axis on histological sections. The heart section schematic shows the positions of the three measurements. Data were calculated from the mean values of three sections per animal (see also Supplementary *Figures S2 and S3*). (*C*) Representative double immunofluorescence images of Cx43 and N-Cadh in heart cross-sections of WT and G609G mice (crop: zoom of the white square in merge). Asterisks mark intercalate discs where Cx43 colocalize with N-Cadh. Arrowheads point to areas with Cx43 lateralization. The graph shows the quantification of Cx43 and N-Cadh colocalization. (*D*) Representative confocal microscopy images of alpha-tubulin (red) in isolated cardiomyocytes from WT and G609G mice with organized and disorganized cytoskeleton patterns. The graph shows quantification of the percentage of cells with an organized cytoskeleton. Numbers in the bars are the ratios of the number of cardiomyocytes with an organized cytoskeleton to the total number of cells analysed for each genotype (see also Supplementary Video S1). Statistical analyses were conducted using two-tailed *t*-test and Fisher’s exact test. *P<0.05; **P<0.01; ****P<0.0001.

We first conducted magnetic resonance imaging (MRI) and histology experiments. MRI data revealed no significant differences in ventricular ejection fraction between WT and G609G hearts (*Figure 1A*). In contrast, G609G mice had a lower cardiac index in both ventricles (*Figure 1A*) and showed thinning of ventricular walls and interventricular septum (*Figure 1B* and Supplementary *Figure S2*). TUNEL staining did not reveal significant between-genotype differences in apoptosis in left and right ventricles (RVs) (data not shown), and echocardiography showed normal fractional shortening and ejection fraction in G609G mice (Supplementary *Figure S3*). G609G mice showed aberrant connexin-43 (Cx43) localization, indicated by significantly below-normal N-Cadherin (N-Cadh)/Cx43 colocalization (*Figure 1C*), in agreement with our previous data in *Zmptse24^−/−^* mice,^9^ HGPS pigs,^11^ and HGPS patients.^9^

We next performed immunofluorescence studies in isolated cardiomyocytes from WT and G609G mice to visualize alpha-tubulin organization. After capturing z-stacks, we classified the level of tubulin-cytoskeleton organization in a blinded manner, as follows: (i) organized, with longitudinal tubulin-cytoskeletal fibres and showing a transverse pattern reflecting the t-tubules; and (ii) disorganized, with no apparent cytoskeleton organization (showing neither longitudinal nor transverse patterns) and containing tubulin tangles. Based on these criteria, the tubulin-cytoskeleton was significantly disorganized in cardiomyocytes from G609G mice (*Figure 1D* and Supplementary Video S1). Tubulin-cytoskeleton disorganization was also observed in cardiac tissue of progeroid heterozygous *LMNA* c.1824C>T Yucatan minipigs (Supplementary *Figure S4*).

**Figure 2:**
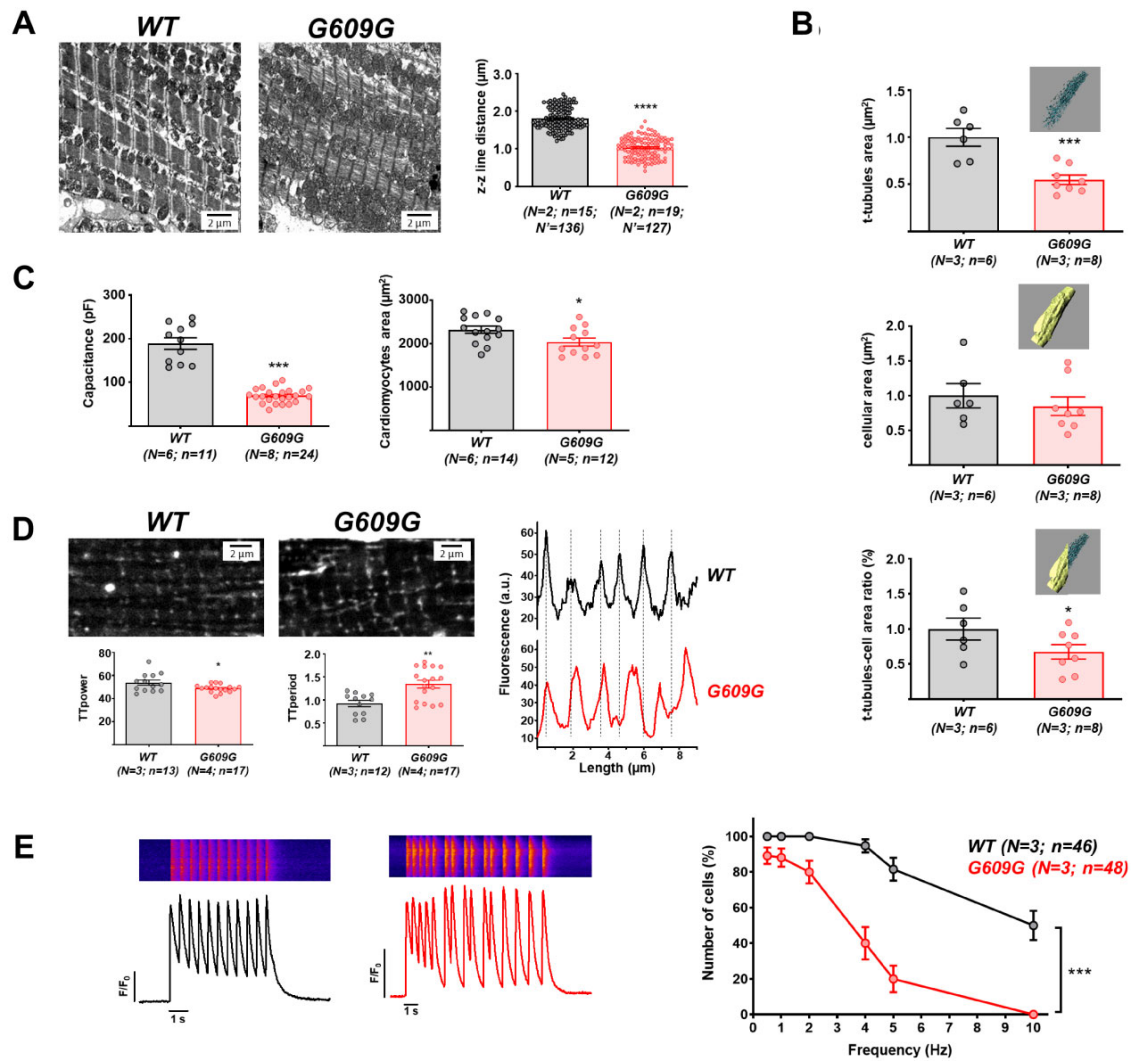
Shorter sarcomeres and disrupted t-tubule system in G609G cardiomyocytes. (*A*) Representative transmission electron microscopy images of cardiac cross-sections from WT and G609G mice, with quantification of sarcomere z–z line distance. (*B*)–(*E*) Cardiomyocytes isolated from WT and G609G hearts were used for the following studies: (*B*) quantification of plasma t-tubule area, cell area, and t-tubule/cell-area ratio by 3D reconstitution and segmentation of cells stained with WGA. Data are shown relative to control group (see also Supplementary Video S2). (*C*) Capacitance and cell area, measured by patch-clamping and 2D microscopy, respectively. (*D*) Representative confocal microscopy images of cells stained with WGA, with quantification of t-tubule organization (TT_power_) and distribution (TT_period_). The graph at the bottom shows the distribution of t-tubules according to the fluorescence profile. (*E*) Calcium-dependent calcium-release measured by field stimulation at different frequencies. Representative fluorescence profiles are shown of Fluo-4-AM recorded at 2 Hz in WT (top) and G609G (bottom). The graph shows quantification of the percentage of cells responding at a 1:1 stimulus: response ratio. Statistical analyses were conducted using two-tailed *t*-test and one-way ANOVA. *P<0.05; **P<0.01; ***P<0.001; ****P<0.0001.

Previous studies in models of dilated cardiomyopathy caused by *LMNA* gene mutations have shown sarcomeric organization defects associated with abnormal activation of the Cofilin 1, ERK1/2, and p38α pathways in the heart.^29–31^ Transmission electron microscopy revealed altered sarcomere ultrastructure in G609G cardiomyocytes, with sarcomere shortening indicated by a reduced distance between z lines (*Figure 2A*). Moreover, western blot analysis showed significantly higher levels of phospho-ERK1/2, phospho-Cofilin 1, and p38α in G609G hearts (Supplementary *Figure S5*). We also analysed cell area by 3D rendering of wheat germ agglutinin (WGA)-stained isolated cardiomyocytes followed by segmentation of t-tubules and plasma membrane. While cell area did not differ between genotypes, the area occupied by the t-tubules and the t-tubule/cell-area ratio were both significantly lower in G609G cells (*Figure 2B* and Supplementary Video S2). Consistent with these findings, patch-clamping revealed a much lower membrane capacitance in G609G cardiomyocytes that was not attributable to cell-area differences detected by 2D microscopy (*Figure 2C*).

Analysis of the WGA fluorescence images with the ImageJ TTorg plugin^32^ revealed t-tubule disorganization in G609G cardiomyocytes (relatively low TTorg parameter values) and a low t-tubule frequency (relatively high TTperiod parameter values); this finding was corroborated by the fluorescence profiles (*Figure 2D*). Taken together, the results presented thus far suggest that the t-tubule membrane and sarcomere are misaligned in G609G cardiomyocytes, which would predict inefficient excitation–contraction (EC) coupling. Consistent with this hypothesis, intracellular calcium transients showed significant alterations in G609G cardiomyocytes, including a slower rise, lower magnitude, and slower recovery, although total calcium release was unaltered (see below). In addition, G609G cardiomyocytes had large numbers of spontaneous calcium transients occurring prematurely in rapid succession, and the percentage of G609G cells able to respond 1:1 (stimulus: response) to rhythmic field stimulation was significantly below normal (*Figure 2E*).

### 3.2 G609G mice show bradycardia and QT prolongation

**Figure 3:**
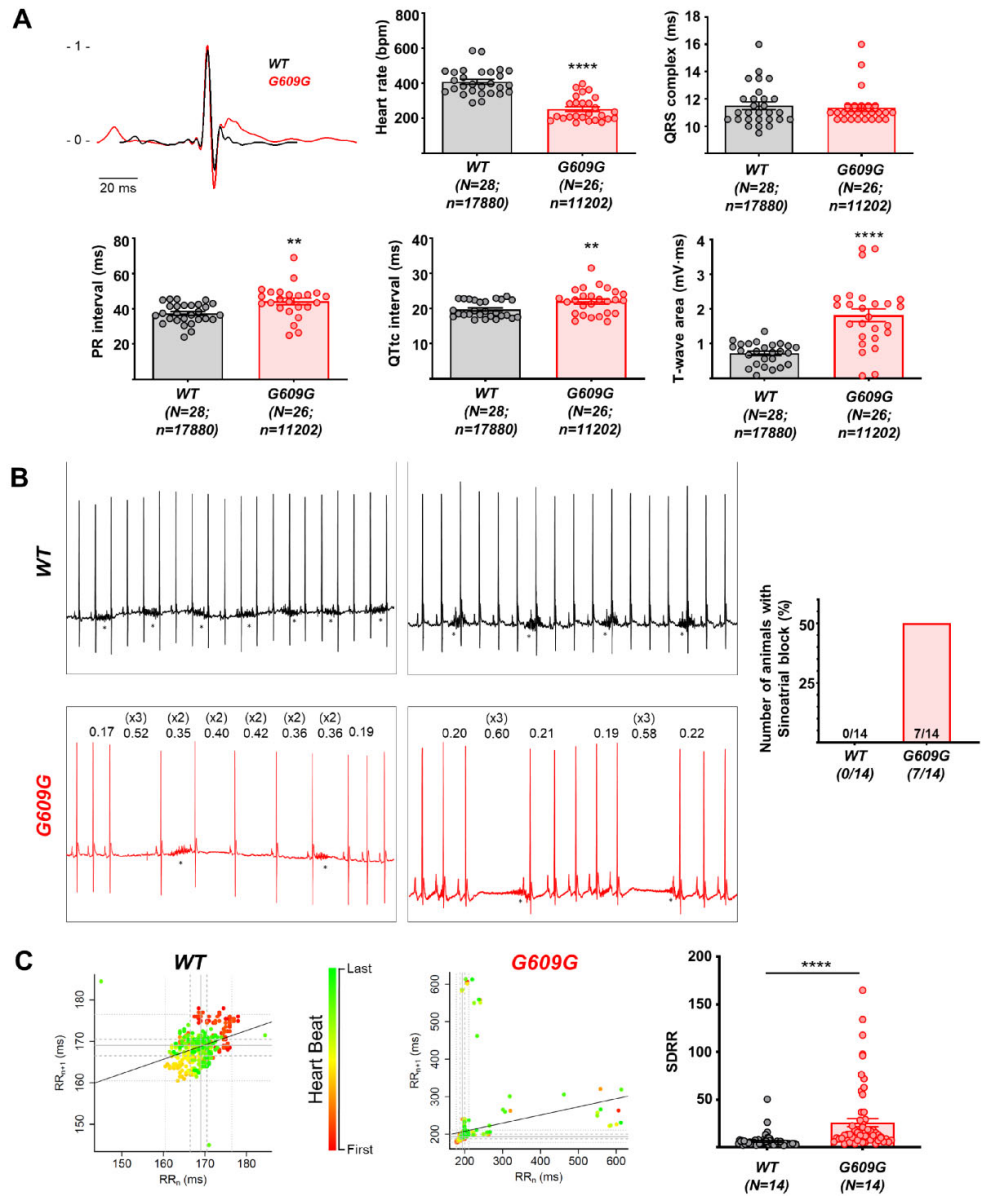
Electrocardiographic analysis shows cardiac repolarization defects and sinoatrial block in G609G mice. (*A*) Extracted averaged ECG lead II from WT (black) and G609G (red) mice, heart rate, QRS, PR, QTtc intervals, and T-wave area in anaesthetized WT and G609G mice. (*B*) Representative ECG recordings, with numbers in the G609G traces showing the RR-interval amplitude. Asterisks indicate the presence of breathing artefacts. The graph shows the incidence of sinoatrial block after 1 min of recording. (*C*) RR_*n*_/RR_*n*__+1_ correlation plots (circle maps) and SDRR in WT and G609G animals after 1 min of recording. Statistical analyses were conducted using two-tailed *t*-test and Fisher’s exact test. **P<0.01; ***P<0.001.

ECG recordings showed significant alterations in heart rate, PR, QT intervals, and T-wave area in G609G mice (*Figure 3A*), suggesting possible defects in action potential (AP) initiation, conduction, and duration (see below). G609G mice also showed frequent pauses in the cardiac rhythm, with loss of P-waves and QRS-complexes (*Figure 3B*), a characteristic phenotype of sinoatrial block. Consistent with these findings, G609G mice had a significantly altered RR circle map and a larger standard deviation of the RR-interval (SDRR) (*Figure 3C*).

### 3.3 Post-repolarization refractoriness in G609G mouse cardiomyocytes

**Figure 4:**
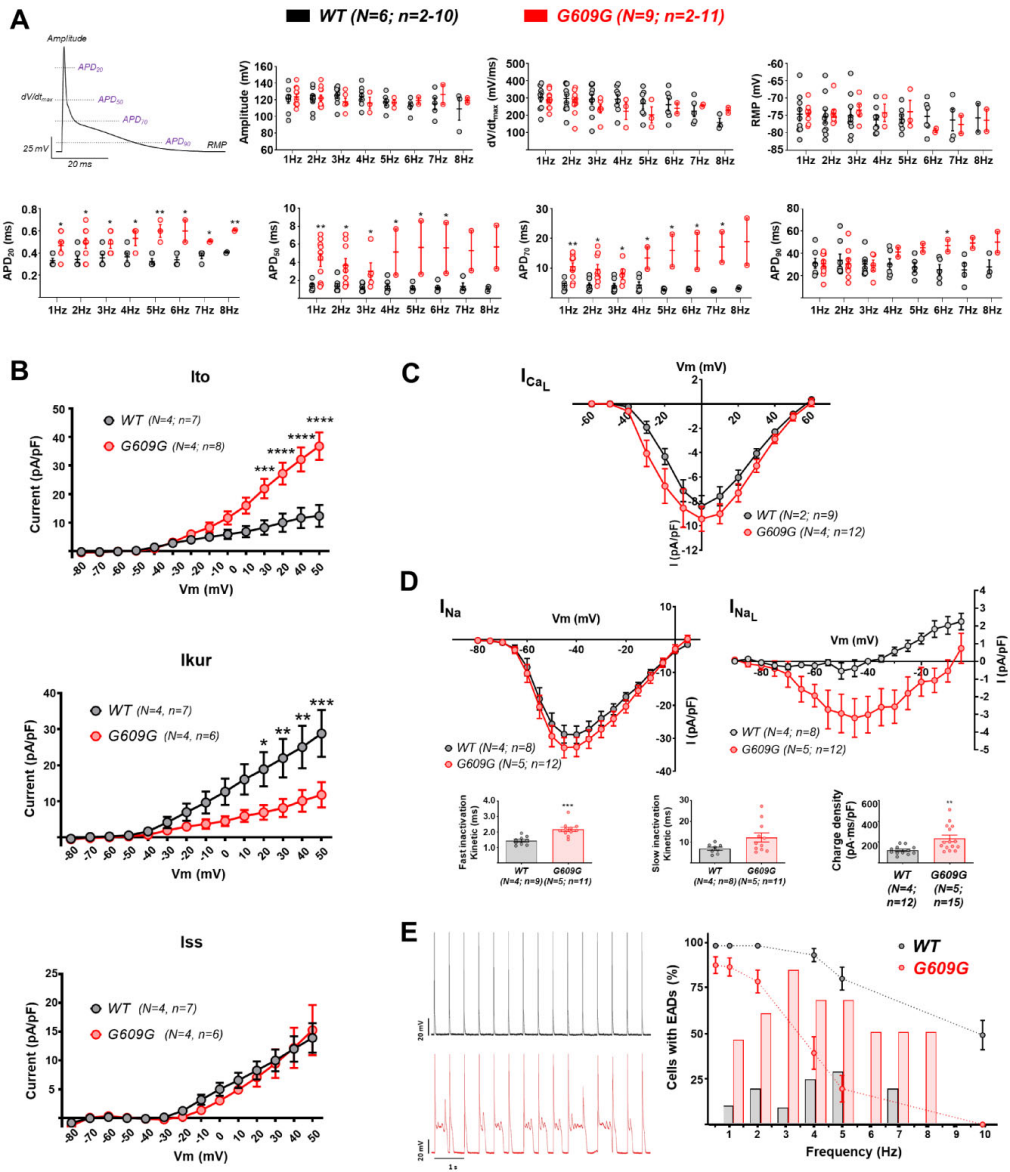
G609G cardiomyocytes exhibit prolonged APD and post-repolarization refractoriness. Isolated WT and G609G mouse cardiomyocytes were analysed by patch-clamp. (*A*) Example of mouse cardiomyocyte AP showing the different parameters analysed. Upper graphs show maximal amplitude of the AP (peak amplitude), maximal d*V*/d*t*, and RMP. Lower graphs show the APD at 20%, 50%, 70%, and 90% repolarization. (*B*) Outward potassium currents *I*_to_, *I*_Kur_, and *I*_ss_. (*C*) L-type calcium current. (*D*) Peak and late sodium currents IV-relationships. The graphs show sodium charge density and inactivation kinetics measured at −40 mV (see also Supplementary *Figures**S7–S9*). (*E*) Correlation between EC-coupling efficiency (dashed line) and the incidence of EADs (bars) at different frequencies. Representative AP recordings are shown at 2 Hz of stimulation in WT (top) and G609G (bottom), reflecting the appearance of EADs in G609G mouse cardiomyocytes. Note that data shown with dashed lines are also shown in *Figure 2E*. Statistical analyses were conducted using two-tailed *t*-test and two-way ANOVA. *P<0.05; **P<0.01; ***P<0.001; ****P<0.0001.

We conducted patch-clamp experiments in isolated WT and G609G cardiomyocytes to investigate whether progerin expression leads to alterations in AP properties at varying pacing frequencies (1–8 Hz). No between-genotype differences were detected in maximum upstroke velocity (d*V*/d*t*), AP amplitude, or resting membrane potential (RMP) at all frequencies tested (*Figure 4A*, top). In contrast, at frequencies between 1 and 3 Hz, there was significant AP duration (APD) prolongation at 20%, 50%, and 70% repolarization in G609G cardiomyocytes, and APD_90_ was prolonged at higher frequencies (*Figure 4A*, bottom). Most importantly, G609G cardiomyocytes were unable to respond in a 1:1 manner to frequencies above 3 Hz (*Figure 4A*, bottom). Moreover, only 2 out of 11 G609G cardiomyocytes (18.2%) showed 1:1 activation at frequencies above 4 Hz, whereas 5 out of 7 (71.4%) WT cardiomyocytes showed 1:1 activation even at these high frequencies (*Figure 4A*, bottom). To correlate these data with abnormal repolarization in some HGPS patients,^4^^,^^9^ we used QT duration data from patients with more than 2 years follow-up.^9^ The analysis showed that although QT prolongation did not reach pathological values in all patients, it was age-dependent and more prevalent in advanced disease stages (Supplementary *Figure S6*).

To investigate the mechanisms underlying the complex frequency-dependent dynamics in G609G cardiomyocytes, we conducted voltage-clamp experiments for the main cardiac ionic currents that operate in mouse ventricles. Compared with WT controls, G609G cardiomyocytes had a higher magnitude transient outward potassium current (*I*_to_) and a lower ultra-rapid current (*I*_Kur_), with no difference in steady-state current (*I*_ss_) (*Figure 4B*). Supplementary *Figure S7* shows a representative example of total outward potassium current in the absence and presence of 200 µM 4-aminopyridine. These results are in agreement with the higher plateau potential in G609G mice with no difference in total APD (see *Figure 4A*, bottom). The magnitudes of L-type calcium (*I*_Ca-L_) and sodium (*I*_Na_) inward currents were similar in G609G and WT cardiomyocytes (*Figure 4C and D* and Supplementary *Figures S8 and S9*). However, *I*_Na_ had slower inactivation kinetics, resulting in higher charge density and higher late sodium current (*Figure 4D*) in G609G cardiomyocytes, which might explain the bradycardia and prolonged refractory period in G609G mice (*Figures 3A and 4A*, bottom). These data are in full agreement with those obtained in the calcium transient experiments presented in *Figure 2E*, which correlate with frequency-dependence APD prolongation and the occurrence of early afterdepolarizations (EADs) (*Figure 4E* and *Table 1*). We also examined the expression level and phosphorylation of Ca^2+^/calmodulin-dependent protein kinase II (CaMKII), which regulates cardiac sodium channels and is affected in heart failure.^33^ Our western blots showed no significant changes in CaMKII expression and phosphorylation when comparing WT and G609G hearts (data not shown).

**Table 1:** Presence of EADs in isolated cardiomyocytes analysed by patch-clamping

	WT		G609G	
Frequency (Hz)	Number positive cells	%	Number positive cells	%
1	1/9	11	5/11	46
2	2/10	20	6/10	60
3	1/10	10	5/6	83
4	2/8	25	2/3	67
5	2/7	29	2/3	67
6	0/6	0	1/2	50
7	1/5	20	1/2	50
8	0/3	0	1/2	50

### 3.4 Chronic treatment with paclitaxel partially rescues the structural and functional alterations in G609G mice

**Figure 5:**
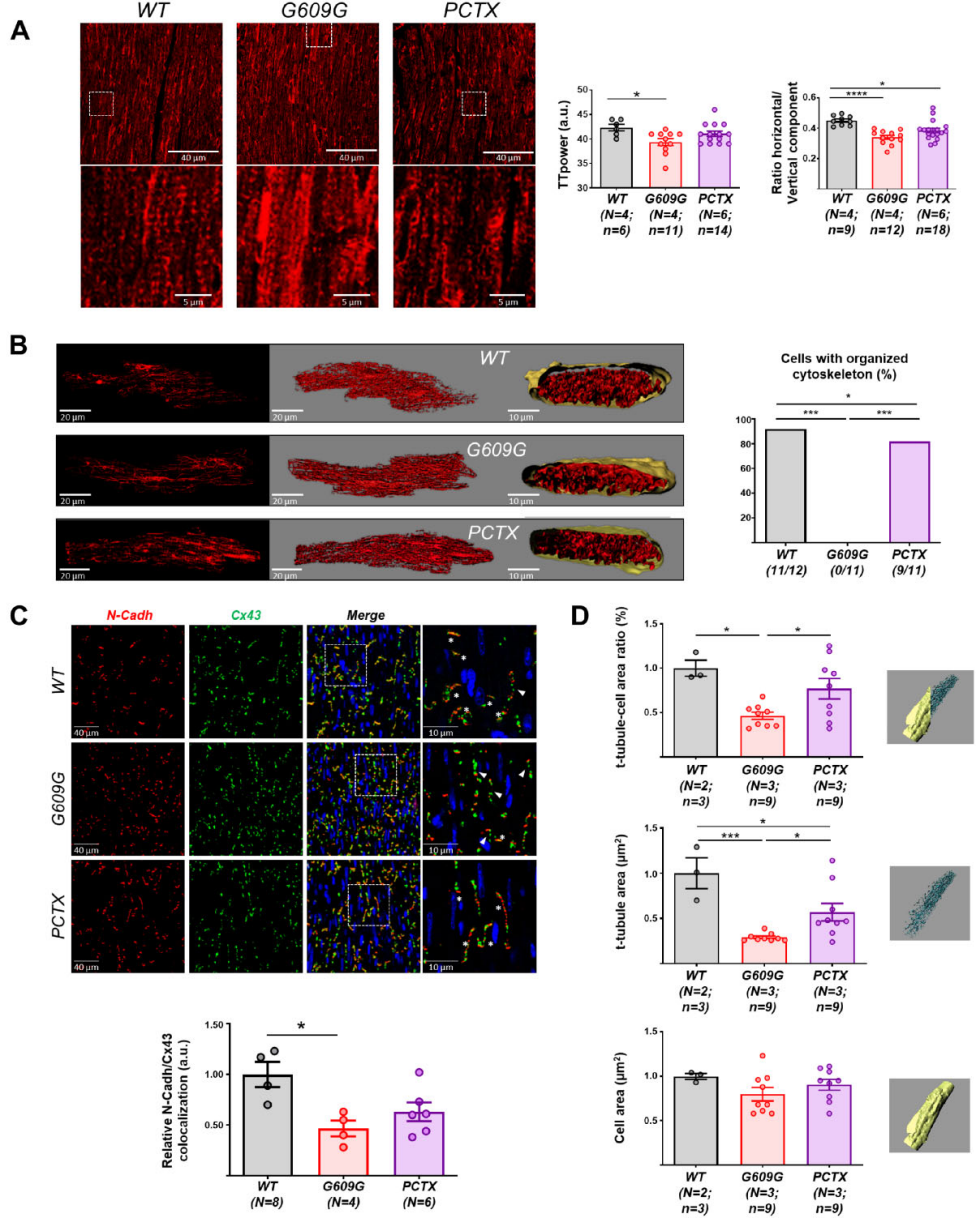
Chronic paclitaxel treatment improves cardiac structural and molecular alterations in G609G mice. In all panels, PCTX refers to G609G mice treated with paclitaxel. (*A*) Representative confocal microscopy images of alpha-tubulin in heart cross-sections (crop: zoomed view of the white boxed area). The graphs show quantification of TT_power_ and the horizontal: vertical component ratio. (*B*) Representative 3D reconstructions of tubulin-cytoskeleton visualized with anti-α-tubulin antibody; the graph shows quantification of the percentage of cells with an organized cytoskeleton. (*C*) Representative immunofluorescence images of Cx43 and N-Cadh in heart cross-sections. The white boxed areas in the merge image are shown at larger magnification in the right. Asterisks mark intercalate discs where Cx43 colocalize with N-Cadh. Arrowheads point to areas with Cx43 lateralization. The graph shows quantification of Cx43 and N-Cadh colocalization. (*D*) T-tubule system analysis by 3D rendering of WGA staining in isolated cardiomyocytes to quantify the t-tubule: cell-area ratio, t-tubule area, and cell area. Data are shown relative to control group. Statistical analyses were conducted using one-way ANOVA and Fisher’s exact test. *P<0.05; ***P<0.001; ****P<0.0001.

**Figure 6:**
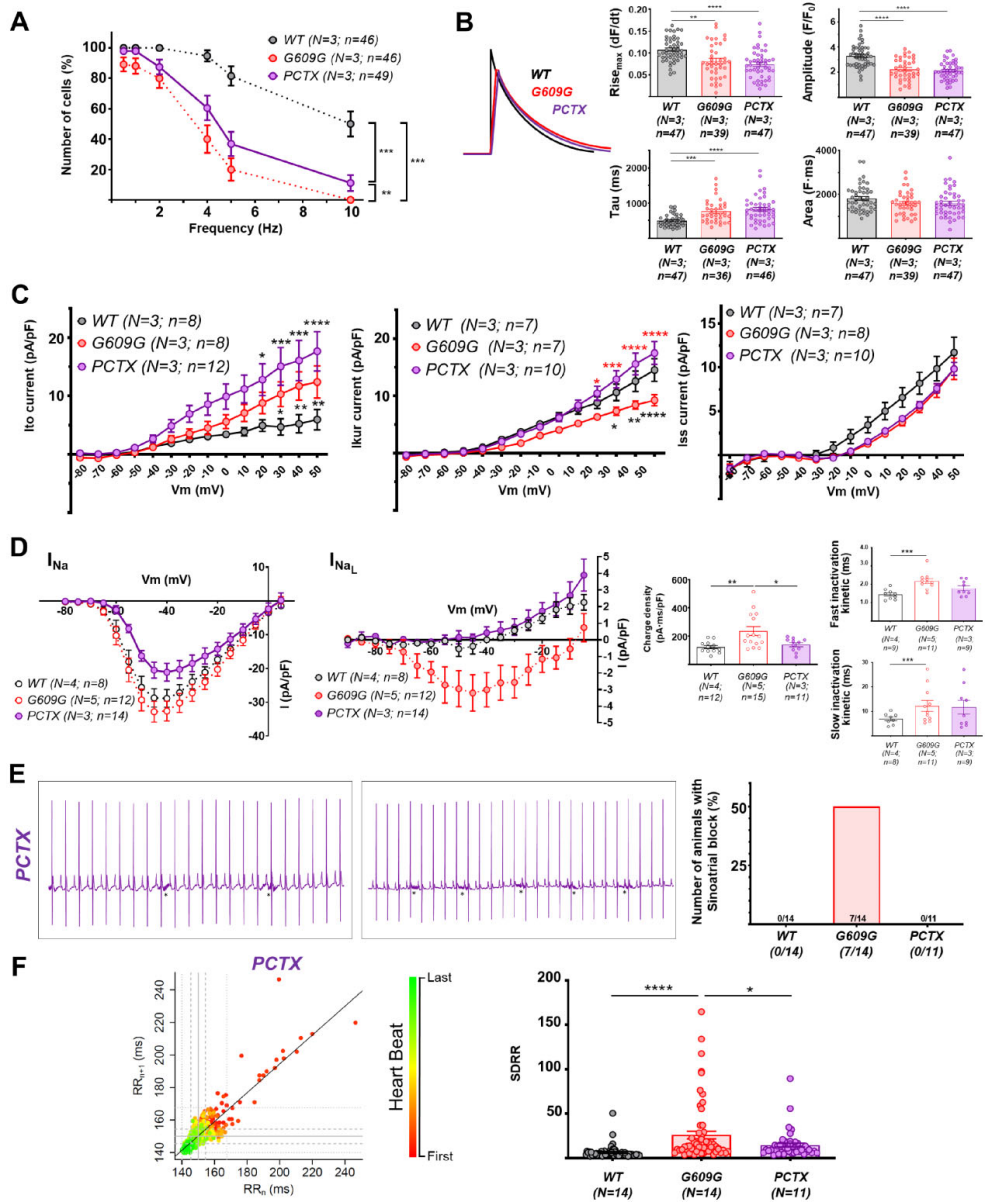
Chronic paclitaxel treatment improves functional cardiac alterations in G609G mice. In all panels, PCTX refers to G609G mice treated with paclitaxel, and WT to WT mice. (*A*) EC coupling analysis in isolated cardiomyocytes to quantify the percentage of cells responding at a 1:1 stimulus: response ratio at different stimulation frequencies. (*B*) Calcium dynamics in response to 0.5 Hz stimulation: maximum rise (Rise_max_), amplitude, tau, and area. The curve plot shows representative calcium pulses. (*C*) Outward potassium currents *I*_to_, *I*_Kur_, and *I*_ss_. (*D*) Peak and late sodium currents IV-relationships. The graphs show sodium charge density and inactivation kinetics measured at −40 mV (see also Supplementary *Figures S7–S9*). (*E*) Representative ECG recordings from the PCTX group. Asterisks indicate the presence of breathing artefacts. The graph shows the incidence of sinoatrial block after 1 min of recording. (*F*) RR_*n*_/RR_*n*__+1_ correlation plots and SDRR quantification during ECG recording. Note that data from WT and G609G shown in (*A*) and (*D*)–(*F*) are also shown in *Figures 2E, 4D, and 3B and C*, respectively, because experiments with the PCTX group were performed together with WT and G609G mice. Statistical analyses were conducted using one-way and two-way ANOVA, and Fisher’s exact test. *P<0.05; **P<0.01; ***P<0.001; ****P<0.0001.

To assess whether tubulin-cytoskeleton disorganization contributes to cardiac alterations in G609G mice, we treated animals chronically with a low dose of paclitaxel (Taxol^®^), which stabilizes microtubules by binding to the β-subunit of tubulin.^34^ To ascertain whether the potential improvement in G609G-treated mice was full or only partial, we included a group of untreated WT mice as a reference for normal phenotype. Analysis of cardiac tissue (*Figure 5A*) and isolated cardiomyocytes (*Figure 5B*) revealed improved cytoskeletal organization in paclitaxel-treated G609G mice compared with untreated controls. These studies confirmed a statistically significant reduction in Cx43/N-Cadh colocalization in G609G compared with WT heart, but differences were not statistically significant when comparing WT and paclitaxel-treated G609G mice, indicating partial normalization of Cx43 localization (*Figure 5C*). Paclitaxel also partially restored t-tubule system density (*Figure 5D*) without changing capacitance (Supplementary *Figure S10*). Functionally, paclitaxel significantly increased the percentage of cells responding to increasingly higher frequencies, although it did not restore the levels seen in WT controls (*Figure 6A*) and did not revert the calcium dynamics found in G609G cells (slower rise, lower magnitude, and slower decrease, but with a similar total calcium release) (*Figure 6B*). Paclitaxel also improved cardiomyocyte electrophysiology by increasing *I*_to_, restoring the magnitude of *I*_kur_ without changing *I*_ss_ (*Figure 6C*) and restoring sodium charge density to WT levels by a decrease in *I*_Na_ with faster inactivation kinetics (*Figure 6D*). Finally, chronically administered paclitaxel completely reverted the sinoatrial block observed on the ECG of G609G mice (*Figure 6E*), and this reversion translated into higher linear correlation between RR-intervals and a significant decrease in the SDRR (*Figure 6F*).

## 4. Discussion

HGPS is an extremely rare genetic disease characterized by cardiovascular alterations, premature aging, and death during adolescence.^3–5^ However, the mechanisms by which progerin expression causes cardiovascular disease remain largely unknown. Here, we investigated progerin-induced cardiac alterations, which have been reported in animal models of progeria and in HGPS patients.^4^^,^^9–11^^,^^22^^,^^35^ Changes in nuclear envelope and structure associated with *LMNA* mutations are known to cause cytoskeletal alterations and therefore lead to defects in cell structure and protein trafficking and function.^17^^,^^29–31^^,^^36^^,^^37^ Consistently, we have shown here that progerin expression in G609G mice was strongly associated with structural alterations to the tubulin-cytoskeleton in the heart, with consequent functional and electrophysiological alterations associated with premature aging (Graphical Abstract). These alterations include increased heart rate variability, sinus bradycardia, sinoatrial block, and PQ prolongation, all significant signs of cardiac conduction system defects, as well as prolonged repolarization in ventricular cardiomyocytes. Some of these changes are consistent with electrical alterations present in the elderly, and repolarization abnormalities have been also described in HGPS patients and animal models.^9–11^

Tubulin-cytoskeletal disruption alters the structure and function of the t-tubule system, leading to defects in EC-coupling and calcium dynamics. Indeed, cardiomyocyte-detubulation has been shown to provoke alterations in EC-coupling and calcium dynamics.^38^ Here, we detected disorganization of the tubulin-cytoskeleton in G609G mouse cardiomyocytes, together with defects in the t-tubule system and misalignment between t-tubules and the Z lines in the sarcomere, all resulting in EC-uncoupling. We also observed tubulin-cytoskeleton disorganization in cardiac tissue of progeroid heterozygous *LMNA* c.1824C>T minipigs. Sarcomere organization, including structure and length, is essential for the heart to contract and provide enough blood to meet the body’s demands. The progerin-dependent alterations in the tubulin-cytoskeleton and sarcomere structure are thus a likely underlying cause of the cardiac function defects in G609G mice revealed by MRI, including a ∼50% decrease in the cardiac index in both ventricles and thinning of the ventricles and septum. These defects are potentially involved in heart failure symptoms in HGPS patients.^3–5^

We previously reported electrophysiological defects in HGPS animal models, including progeroid *Zmptse^−/−^* mice,^9^ G609G mice,^10^ and *LMNA* 1824 C>T knockin minipigs.^11^ Here, we also found cardiac electrophysiological alterations in G609G mice, including bradycardia, prolonged PR and QT intervals, and Cx43 mislocalization. Moreover, to our knowledge, we report here for the first time that 50% of G609G mice exhibit ECG evidence of premature aging,^39^ with overt alterations in heart rate variability, inappropriate sinus bradycardia, sinus pause, sinoatrial block, and atrio-ventricular delay (PR prolongation). In human aging, bradyarrhythmias are manifestations of an altered pacemaking and cardiac conduction system whose progression depends on the presence and severity of associated coronary or hypertensive heart disease.^40^ Cx43 mislocalization may contribute to the occurrence of these altered conduction patterns in G609G mice as also documented in progeroid *Zmpste24^−/−^* mice^9^ and pigs.^11^ Future studies are warranted to assess whether connexin40 mislocalization might also contribute to conduction system defects in progeroid hearts. Altered electrical activation also occurred over a wide range (1–10 Hz) of stimulus frequencies in G609G cardiomyocytes, which responded intermittently to repetitive stimulation because of APD prolongation and EAD formation, a behaviour that was evident from measurements of AP and calcium transients. The most likely mechanism underlying this behaviour and the sinoatrial block is increased sodium inward charge due to slower *I*_Na_ inactivation. In G609G mouse ventricular cardiomyocytes, increased plateau duration and APD prolongation at frequencies above 3 Hz was accompanied by increased refractoriness and EADs.^41^ APD prolongation increases the vulnerability to block and initiation of re-entrant impulses in the atria or ventricles and has been linked to increased risk and lethality of ventricular arrhythmias during ischaemia.^42^ Ventricular repolarization abnormalities like T-wave alternans or ischemic-related alterations can also increase the risk of ventricular arrhythmia. In fact, we have previously documented an increase in premature ventricular complexes in progeroid *Zmpste24^−/−^* mice associated with overt and age-dependent T-wave abnormalities compared with controls.^9^ Importantly, such T-wave abnormalities follow the same age-dependent pattern in patients, and although not documented in our series,^9^ this may also prone patients to higher risk of ventricular arrhythmia upon ischaemia or bradycardia in late stages of the disease.^9^^,^^35^^,^^43^ Our current study provides further underlying ionic mechanisms explaining a higher risk of EADs in the presence of an increased sodium inward charge due to slower *I*_Na_ inactivation. Together, the above mechanism and prolongation of repolarization explain the intermittent sinoatrial and ventricular block patterns in G609G mice.^41^ Alterations in sodium current inactivation kinetics have also been shown to occur in ischemia^43^ and in Brugada syndrome and long QT syndrome.^44^^,^^45^ Moreover, the observed alterations in outward potassium currents in the G609G mouse, namely increase in *I*_to_ and decrease in *I*_Kur_, would also contribute to the presence of sustained depolarization at early phases of repolarization with higher incidence of EADs.^46^^,^^47^^,^^48^ As previously suggested,^41^ all these changes could explain why G609G mouse cardiomyocytes are unable to respond properly to frequencies higher than 3 Hz.

Cardiac ion channels are highly conserved between humans and mice. Nevertheless, there are significant differences regarding the number of ion channel types that contribute to the cardiac AP,^46^^,^^49^ making it difficult to translate mouse findings to the clinic. Our results in human induced pluripotent stem cell-derived cardiomyocytes (hiPSC-CMs) from a HGPS patient show electrophysiological alterations similar to those found in the G609G mouse model (Supplementary *Figure S11*). Specifically, we find that the functional consequences of progerin expression are similar in hiPSC-CMs and mouse cardiomyocytes, since both had longer APDs than their controls, and would be unable to respond to stimulation at frequencies above a critical threshold because of prolonged repolarization. This is consistent with the presence of age-dependent abnormal QT values in some HGPS patients.^4^^,^^9^ Other mechanisms or less advanced disease stages may counteract this phenotype in patients with normal QT values.

We found that sarcomeric organization defects in the G609G heart are associated with higher levels of phospho-ERK1/2, phospho-Cofilin 1, and p38α. These data are consistent with previous studies in dilated cardiomyopathy models caused by *LMNA* gene mutations, which showed that sarcomeric alterations are also associated with abnormal activation of these signalling proteins.^29–31^ Remarkably, the p38α inhibitor ARRY-371797 improved left ventricular dilation and fractional shortening in mutant Lmna (H222P/H222P) mice,^31^ and this compound is currently under assessment in Phase 2 and 3 clinical trials in patients with *LMNA*-related dilated cardiomyopathy (ClinicalTrials.gov Identifiers: NCT02351856, NCT03439514). Future studies are thus warranted to assess whether pharmacological inhibition of ERK1/2, Cofilin 1, and p38α ameliorates cardiac alterations in HGPS. Given the cytoskeletal disorganization observed in the G609G mouse heart, we treated progeric animals with paclitaxel, a drug that stabilizes microtubules.^34^ Chronic low-dose treatment with paclitaxel induced tubulin-cytoskeleton stabilization in the G606G mouse heart, increased cytoskeletal organization, improved t-tubule structure and EC-coupling efficiency, and reverted the altered cardiac and cardiomyocyte electrophysiology. Future studies are needed to ascertain why treatment of G609G mice with paclitaxel improved ionic currents and t-tubular system but did not affect calcium dynamics. Interestingly, chemical inhibition of NAT10, of which tubulin is a known substrate, ameliorated microtubule organization and HGPS cellular defects.^50^^,^^51^ We surmise that our findings also open a new alternative or complementary approach to reverse or mitigate the cardiac phenotype of HGPS patients. Importantly, paclitaxel is widely used in cancer research and in the clinic^52^ and is therefore potentially translatable to the treatment of cardiac diseases associated with laminopathies. Indeed, paclitaxel has been safely administered to cancer patients with pre-existing cardiac alterations.^53^ Other microtubule-stabilizing agents also have potential for clinical development in cardiac diseases.^52^^,^^54^

In conclusion, we have shown that progerin expression in G609G mice provokes cardiac tubulin-cytoskeleton alterations and functional and electrophysiological anomalies associated with premature aging, including cardiac conduction system defects, and prolonged repolarization in ventricular cardiomyocytes. Chronic low-dose treatment of G609G mice with the microtubule stabilizer paclitaxel mitigates structural alterations and cardiac conduction system defects in G609G mice. Stabilizing the tubulin-cytoskeleton thus might not only provide a new route to reverse or decrease the cardiac phenotype of HGPS patients; it also might improve cardiac electro-mechanical function caused by structural anomalies inherent to normal myocardial aging.

## Supplementary material

Supplementary material is available at *Cardiovascular Research* online.

## Authors’ contributions

A.M., A.M.d.R., D.F.-R., J.J., and V.A. designed the research studies; A.M., J.J.D.-L., Y.B., V.F., C.G.-G., D.P.-B., A.A., and P.G. conducted experiments; A.M., J.J.D.-L., D.P.-B., A.A., and D.F.-R. acquired and analysed data; C.G.-G. and M.J.A.-M. generated and maintained mice; A.M., J.J., and V.A. coordinated the study; and A.M., D.F.-R., J.J., and V.A. wrote the article.

## Supplementary Material

cvab055_Supplementary_Data

## Notes

Translational perspective Mutations in the human *LMNA* gene cause a group of diseases called laminopathies, including the premature aging disorder HGPS. This rare disease is provoked by progerin, a truncated variant of lamin A, which is also expressed at low levels in cells during normal aging. Progerin expression in mice is strongly associated with structural alterations in the cardiomyocyte’s tubulin-cytoskeleton, with consequent cardiac functional and electrophysiological alterations associated with premature aging. Chronic treatment with low dose of paclitaxel partially corrects progerin-induced cardiac alterations. Stabilizing the tubulin-cytoskeleton might therefore provide a new route to reverse or decrease the cardiac phenotype of HGPS patients.
